# Deep Learning for MRI Segmentation and Molecular Subtyping in Glioblastoma: Critical Aspects from an Emerging Field

**DOI:** 10.3390/biomedicines12081878

**Published:** 2024-08-16

**Authors:** Marta Bonada, Luca Francesco Rossi, Giovanni Carone, Flavio Panico, Fabio Cofano, Pietro Fiaschi, Diego Garbossa, Francesco Di Meco, Andrea Bianconi

**Affiliations:** 1 Neurosurgery Unit, Department of Neuroscience, University of Turin, Via Cherasco 15, 10126 Turin, Italy; marta.bonada@unimi.it (M.B.); fabio.cofano@unito.it (F.C.); diego.garbossa@unito.it (D.G.); 2 Department of Neurosurgery, Fondazione IRCCS Istituto Neurologico Carlo Besta, Via Celoria 11, 20133 Milan, Italy; giovanni.carone@unimi.it (G.C.);; 3 Department of Informatics, Polytechnic University of Turin, Corso Castelfidardo 39, 10129 Turin, Italy; lucafrancesco.rossi@polito.it; 4 Division of Neurosurgery, Ospedale Policlinico San Martino, IRCCS for Oncology and Neurosciences, Largo Rosanna Benzi 10, 16132 Genoa, Italy; pietro.fiaschi@unige.it; 5 Department of Neuroscience, Rehabilitation, Ophthalmology, Genetics and Maternal and Child Health, University of Genoa, Largo Rosanna Benzi 10, 16132 Genoa, Italy

**Keywords:** artificial intelligence, deep learning, magnetic resonance imaging, glioblastoma, segmentation, molecular data, clinical applicability

## Abstract

Deep learning (DL) has been applied to glioblastoma (GBM) magnetic resonance imaging (MRI) assessment for tumor segmentation and inference of molecular, diagnostic, and prognostic information. We comprehensively overviewed the currently available DL applications, critically examining the limitations that hinder their broader adoption in clinical practice and molecular research. Technical limitations to the routine application of DL include the qualitative heterogeneity of MRI, related to different machinery and protocols, and the absence of informative sequences, possibly compensated by artificial image synthesis. Moreover, taking advantage from the available benchmarks of MRI, algorithms should be trained on large amounts of data. Additionally, the segmentation of postoperative imaging should be further addressed to limit the inaccuracies previously observed for this task. Indeed, molecular information has been promisingly integrated in the most recent DL tools, providing useful prognostic and therapeutic information. Finally, ethical concerns should be carefully addressed and standardized to allow for data protection. DL has provided reliable results for GBM assessment concerning MRI analysis and segmentation, but the routine clinical application is still limited. The current limitations could be prospectively addressed, giving particular attention to data collection, introducing new technical advancements, and carefully regulating ethical issues.

## 1. Introduction

Gliomas, and among them, glioblastoma multiforme (GBM), stands as the most common and deadliest form of primary brain tumor in adults, characterized by its aggressive growth and poor prognosis. According to the Central Brain Tumor Registry of the United States, GBM accounts for 14.6% of all primary brain tumors and an alarming 48.6% of primary malignant brain tumors, with a median survival rate of approximately 15 months post-diagnosis [1]. The complexity of GBM’s treatment lies in its highly heterogeneous nature, both genetically and in its response to treatment, necessitating personalized therapeutic approaches [2,3,4]. In this context, magnetic resonance imaging (MRI) emerges as a cornerstone in the diagnosis, treatment planning, and follow-up of GBM, providing detailed insights into the tumor’s location, size, and interaction with surrounding brain structures. The segmentation of GBM from MRI scans, a critical step in delineating the tumor boundaries, is traditionally performed manually by expert radiologists. This process, however, is not only time-intensive but also prone to variability, underlining the urgent need for more efficient and standardized approaches [5].

Recent advances in artificial intelligence (AI), particularly deep learning, have shown promising potential to address these challenges [6]. Deep learning models, trained on vast datasets of annotated MRI scans, can automate the segmentation process, offering not just speed and efficiency but also the promise of reducing human error [7]. This technological leap could revolutionize the clinical management of GBM, from enhancing diagnostic accuracy to informing the surgical strategy and assessing treatment response. Yet, the path to integrating deep learning into clinical practice is fraught with obstacles. These range from technical challenges, such as the need for large, diverse training datasets and the management of imaging variability, to broader concerns around algorithm validation, integration into clinical workflows, and ethical considerations.

Taking these considerations as a premise, in this article, we intend to provide a comprehensive overview of the current state of deep learning applications in MRI segmentation and molecular subtyping for glioblastoma, critically examining the limitations that hinder their broader adoption in clinical practice and molecular research. Considering the breadth of topics covered, we introduce Table 1 to summarize the various points and facilitate readability.

## 2. Technical Challenges

### 2.1. MRI Imaging Heterogeneity

Unlike the CT scanner, MRI is characterized by scanner-dependent variation in image signal intensity related to variability in time points, vendors, magnetic field strengths, and acquisition settings [8,9,10]. Radiomic features are highly sensitive to the level of the signal intensity in the image and, thus, non-biological alterations should be removed [11]. Therefore, to obtain reproducible results in radiomic analysis, MRI signal intensity must be standardized. The standardization should obtain an adequate range and distribution of voxel intensity in the different MRIs [12]. Nowadays, there is no general consensus concerning the most reliable standardization approach to adopt.

AI in the field of medical imaging faces unique challenges due to the innate complexity and diversity of medical data [11]. AI models are intrinsically designed to discern and learn from patterns within their training data. However, these patterns may not purely reflect the biological and pathological information of interest, but also the methodological biases present in the data acquisition process.

When training datasets are predominantly composed of MRI scans from a single medical center, the AI model may inadvertently prioritize the specific characteristics of that center’s imaging protocol over the more crucial pathological features of the tumor itself. This situation leads to an overfitting, where the model performs exceptionally well on the training data due to its familiarity with the protocol-specific nuances but fails to generalize this performance to new, unseen data from other centers with different imaging protocols. This issue is exacerbated in the context of gliomas, a highly heterogeneous group of brain tumors, both biologically and morphologically. The heterogeneity is an essential aspect of the disease that AI models must capture to generalize effectively across different patient populations. Therefore, to develop AI models that are robust and generalizable, it is essential to train them on diverse, multi-center datasets that encompass the broad spectrum of imaging techniques and the varied appearances of glioblastoma [13].

For such a reason, for MRI radiomics analysis, a key challenge is to ensure repeatability and reproducibility of the results in the removal of scanner-dependent signal intensity changes [12]. In fact, intensity standardization helps in making the evaluations agnostic to acquisition specifications and allows us to create more reliable models. An interesting tool for MRI harmonization is ComBat, a statistical normalization method for batch-effect correction in genomics that shows promising results in removing scanner-dependent information from extracted features when applied to radiomics [9,10]. Marzi et al. [14] further extended this idea by proposing a harmonizer transformer, an implementation of ComBat allowing its encapsulation as a preprocessing step of a machine learning pipeline, sensitively reducing site effects.

Yet, implementing harmonization in an operative procedure based on the information provided has been shown to have a serious effect on the level of repeatability and redundancy of features [15]. For such a reason, several studies suggest that rescanning data provides the opportunity to assess radiomic feature reproducibility on images from the same patient acquired within a short time delay, despite the minimal modification that a tumor can present within several days [10,15].

### 2.2. Missing MRI Sequences

In clinical practice, obtaining multiple sequences is time-consuming and expensive [16]. Moreover, MRI examinations may vary among institutions because of different acquisition protocols and/or different hardware with unequal resolution capabilities [17].

Nevertheless, for brain tumor segmentation, multi-contrast MRI modalities such as T1, T2, Fluid-Attenuated Inversion Recovery (FLAIR), and T1 Contrast-Enhanced (T1CE) play an essential role in collecting the informative features [18,19]. Utilizing multimodal data through concatenating multiple MR images as inputs for any machine learning method has demonstrated a proficiency in enhancing the semantic segmentation performances of brain tumors [20,21]. More specifically, each imaging modality enables the deep learning convolutional networks to extract and learn reciprocal knowledge to segment different subregions of glioblastoma. For example, peritumoral edema appears as a hyperintensity area in T2 and FLAIR images, while enhancing tumor is highlighted with hyperintensity in T1CE image [22]. Unfortunately, it is usually required to have the complete four image modalities as inputs (i.e., T1, T2, FLAIR, and T1CE). Thus, the problem of missing modalities from MRI examinations leads to challenges for the segmentation task [23,24]. The complexity increases even more with postoperative segmentation, where the standard sequences listed above should be supplemented with diffusion/ADC maps to better characterize residual areas, which can often be confused with normal postoperative barrier damage, edema changes, or possible vascular alteration.

To tackle this problem, the artificial synthesis of missing target modalities from one or more available modalities has recently attracted increasing attention [25]. However, it remains challenging to achieve accurate segmentation results from the synthesized images [16]. In fact, there is a gap between the synthesized and real target modalities, thus causing the segmentation based on synthesized images to perform worse [26]. Moreover, the model becomes more complex and much deeper than the original independent model and it has a higher risk of overfitting [16]. This issue requires to be addressed with more effective regularization methods to maintain the performance during testing. However, the regularization of these models has rarely been explored in existing works.

Eijgelaar et al. [24] introduced a training method that uses sparsity to enhance model outcomes when working with partial clinical datasets. However, even with these modifications, the highest performance levels were attainable only with the full complement of sequences. In fact, each diagnostic modality, whether it is a variant of MRI, CT, or another type of scan, inherently provides unique information. Consequently, any method that tries to compensate for missing information is essentially attempting to mitigate the impact on the algorithm’s effectiveness [26].

**Figure 1:**
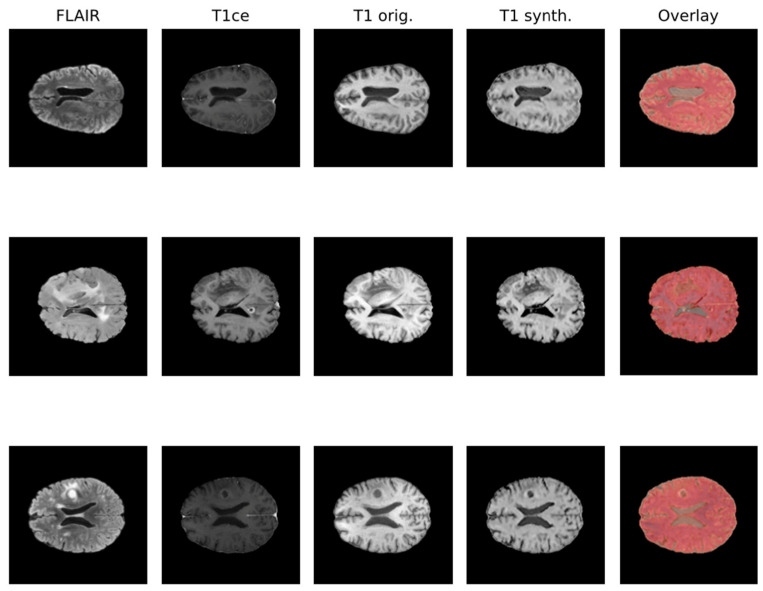
Qualitative comparison of synthesized T1 scans from BraTS 2021 dataset using the IMT technique.

Due to the rise in deep learning, image synthesis within the same modality (such as intramodalities: e.g., from MRI to CT) and across modalities (intermodality: e.g., from FLAIR MRI to T1ce MRI), which entails artificially reconstructing missing sequences from available ones, has attracted considerable attention. This area is emerging as a vibrant and promising research domain. In Figure 1, the IMT model proposed by Osman et al. [27], which was able to generate an accurate synthesis result by generating the missing modalities, is shown.

Various network architectures have been proposed for such tasks in medical imaging within the last few years, but three main backbone models achieved the best results: autoencoder, U-Net, and GAN, with the first starting to lose pace compared to the others [26]. Some studies specifically tackled the problem of brain MRI intramodality synthesis. Yang et al. [28] proposed a method to perform image modality translation (IMT) by leveraging conditional generative adversarial networks (cGANs), whose generator follows the U-Net shape by adding skip connections between mirrored layers in the encoder–decoder network and whose discriminator is derived from a PatchGAN classifier. Osman and Tamam [27] instead implemented a U-Net model aimed at learning the non-linear mapping between a source image contrast to a target image contrast.

Generative adversarial networks (GANs) [29,30] are a relatively new type of DL model that have received much attention because of their ability to generate synthetic images. GANs are trained using two neural networks—a generator and a discriminator. The generator learns to create data that resemble examples contained within the training dataset, and the discriminator learns to distinguish real examples from the ones created by the generator [17]. The two networks are trained together until the generated examples are indistinguishable from the real examples. For such reason, from their conception, GANs have found many applications in medical imaging [31,32].

Another interesting example comes from the Tumor Image Synthesis and Segmentation Network (TISS-Net), a dual-task architecture for end-to-end training and inference, where the synthesis and segmentation models are learned synergistically with several novel high-level regularization strategies [16]. TISS-Net leverages not only a GAN-like architecture comprising a dual-task generator and a dual-task segmentor, but exploits specific domain knowledge while structuring the learning phase, leading to what is known as segmentation-aware target modality image synthesis, where a coarse segmentation is used as an auxiliary task to regularize the synthesis task, and a tumor-aware synthesis loss with perceptibility regularization is introduced to generate segmentation-friendly images in the missing modality. This allows for the improvement and further refinement of the image quality around the tumor region and to reduce the high-level domain gap between synthesized and real target modality images [16].

Another promising approach to overcome the limitation of potential missing sequences is knowledge distillation (KD), which utilizes a teacher–student model to compress model architecture from a cumbersome network to a compact one [18]. In medical image analysis domain, especially in brain tumor segmentation, the KD is used to transfer complete multimodal information from the teacher network to a unimodal student network [33]. However, this is a two-stage approach which requires a training phase for the teacher network with full image modalities. Afterwards, the information is transferred to the student network that utilizes limited modalities. This results in additional training costs and extra time to generate the pre-trained model. Moreover, the teacher might need to be updated or fine-tuned during the training of the student [34]. Choi et al. [18] generated a single-stage-learning knowledge distillation algorithm for brain tumor segmentation. In this case, both models are trained simultaneously using a single-stage knowledge distillation algorithm.

### 2.3. Deployment Issues

Deep learning (DL) recently provided promising results in medical imaging segmentation [35,36]. Nevertheless, the deployment of the available models poses a substantial challenge, mainly related to their computational footprint. In fact, most DL-enabled studies are highly demanding in terms of both energetical and computational resources and such complexities make them very difficult to deploy, especially in tightly controlled clinical scenarios [37].

Moreover, memory constraints in deep learning accelerator cards have often limited training on large 2D and 3D images due to the size of the activation maps held for the backward pass during gradient descent [38]. Two methods are commonly used to manage these memory limitations: the downsampling of images to a lower resolution and/or the breaking of images into smaller tiles [39,40]. Tiling is often applied in the case of large images to compensate the memory limitations of the hardware [41]. In particular, fully convolutional networks can be taught to be translation-invariant and are a natural fit for tiling methods as they can be trained on images of one size and perform inference on images of a larger size by splitting them into smaller sections and thus performing on the smaller tiles [42]. Nevertheless, tiling methods are used primarily to limit the impact of insufficient memory but they usually do not improve the predictive power of the system [38].

The process of quantization is essential to reduce the memory burden during the time of inference [37]. After the process of quantization, a high precision model is reduced to a lower-bit-resolution model (low-precision floating-point or integer quantization are common choices), thus reducing the size of the model and exploiting SIMD or MIMD computations to make inference faster [43].

In the literature, some models of quantization have been created to reduce computational requirements while keeping the segmentation performance stable, like the one by Thakur et al. [37]. In this light, both quantization-aware training and post-training optimizations present themselves as promising approaches for enabling the execution of advanced DL systems on plain commercial-grade GPUs. Therefore, they contribute to the possible spread of DL-based segmenting applications in clinical environments despite the frequent lack of advanced technological support.

Certainly, an increased access to memory remains essential to make a further step in this direction [43]. This should include both improvements in hardware and computing techniques, such as model parallelism [44] and data parallelism [45].

### 2.4. Performance Evaluation

AI segmentation performance is gauged against a reference known as the ground-truth, which, in clinical contexts, is often established via manual segmentation by one or more professional radiologists. Various studies have highlighted the subjective nature of reference standards based on radiologists’ assessments, noting that model performance can fluctuate when trained on different ground-truths [46].

Due to the intrinsic nature of the medical segmentation task, it is hard to consider different ground-truth strategies other than the manually performed labeling. For such reason, common measuring approaches leverage human error subjectivity mitigation trough the averaging of multiple manual segmentations. Annotation averaging is usually performed through tools such as STAPLE, which is based on expectation–maximization and probabilistically corrects noise elements such as outliers or false positives. Usually, this approach is similarly performed for the AI model, exploiting what is known as cross-validation.

**Figure 2:**
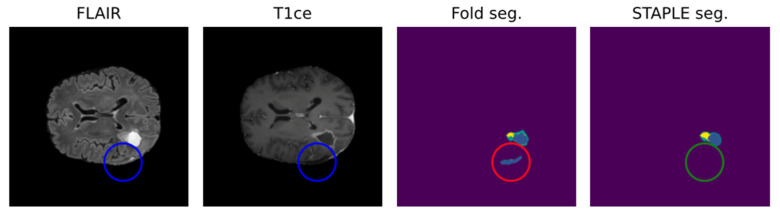
Positive effect of STAPLE fusion for resection cavity segmentation [47]. Results obtained from the fivefold cross-validation process (fold seg.) are merged by the STAPLE algorithm to obtain a final result (STAPLE seg.). The figure shows, as an example, how the STAPLE convergence is able to recognize oversegmentation of a hypointense region misclassified as resection cavity (blue: cavity; yellow: enhancing; green: whole).

Leveraging multiple trained models to aggregate inferences through STAPLE enhances the robustness of AI predictions. Figure 2 shows that STAPLE was able to correct a misclassification case of the resection cavity. Metrics like the Dice Score and Hausdorff distance percentiles are then used to assess the AI model’s true capabilities comprehensively.

Nonetheless, while the significance of human annotations has been widely recognized, the actual process of annotation has received less scrutiny. For instance, Zając et al. [47] highlighted the inherent limitations present in the creation of human-labeled annotations for medical datasets. Sylolypavan et al. [48] also discussed how inherent biases, judgments, and errors from experts could influence AI-driven decision-making in clinical settings.

With the goal of trying to overcome these limitations, some proposals to shift from supervised segmentation tasks to unsupervised ones in the medical environment have been presented. Aganj et al. [49] suggested a whole segmentation approach based on the local center of mass by grouping pixels iteratively in 3D MRIs, which achieved interesting results. Instead, Kiyasseh et al. [50] tackled the problem from an even wider perspective, proposing a novel framework for evaluating clinical AI systems in the absence of ground-truth annotations theoretically capable of identifying unreliable predictions and of assessing algorithmic biases. A recent study from Yale University introduced a whole framework for unsupervised segmentation [51], which exploits image-specific embedding maps and hierarchical dynamic partitioning at different levels of granularity. These demonstrate an improvement ranging from 10% to 200% on Dice coefficient and Hausdorff distance with respect to previous unsupervised proposals.

Even though the performances achieved do not reach the results from supervised training, there is an undeniable surge in proposals targeting the principal challenges of data labeling in recent years. This trend is paving the way for novel approaches in AI-based model design, specifically tailored for clinical application.

## 3. Application to a Real-Word Scenario

### 3.1. Limited Number of Patients

One of the major limitations in the successful translation of AI algorithms into common practice is the limited number of patients included in each study, with a mean of 148.6 patients for each study and a median of 60.5 [52]. Moreover, clinical translation has been significantly hampered due to limited available annotated datasets and decreased performance of algorithms on geographically distinct validation datasets [53,54]. This issue remains in an essential step to obtain an effective training of the algorithm and to lower the risk of overfitting [52].

Paradoxically, the application of complex and sophisticated DL algorithms for segmentation underperforms older ML methods when small datasets are used (n ≤ 15 patients) [55].

As a first step, the creation of single-center datasets is more suitable to the clinical imaging protocols of the hospital and the patient cohort on site [52]. Nevertheless, the most appealing perspectives are data sharing agreements, the development of image databank consortiums (MIDRC, TCIA, BraTS), and federated learning [56,57].

Since 2012, the Brain Tumor Segmentation (BraTS) challenge has implemented and increased the role of ML in glioma MRI evaluation. The focus has been centered on the evaluation of state-of-the art methods for tumor segmentation, the classification of the lesion and, more recently, the prediction of prognosis [58,59].

Additionally, some informatic tools can be applied to limit the impact of data scarcity on the algorithm performance. Among these tools, the application of transfer learning (TL) has been previously explored and applied [46]. TL derives from the cognitive conception that humans can solve similar tasks by exploiting previously learned knowledge, with such knowledge being therefore transferred across similar tasks to improve performances on a new one. Current transfer learning techniques in medical imaging implement knowledge transfer from natural imaging. Overall, two main paths to apply transfer learning have historically been delineated: feature extractor and fine-tuning. The main difference lies in the fact that the first freezes the convolutional layers whereas the latter updates parameters during model fitting. Nonetheless, even if some progress is achieved, the knowledge transferred between the two areas can either be insufficient for achieving promising results in the medical task or make the transfer process quite unpredictable [60]. A recent study by Bianconi et al. [46] applied transfer learning from the preoperative brain tumor segmentation task to postoperative segmentation by fine-tuning the model, showing interesting results and promising multi-site generalization leveraging their structural closeness in the knowledge domain.

### 3.2. Data Quality

**Figure 3:**
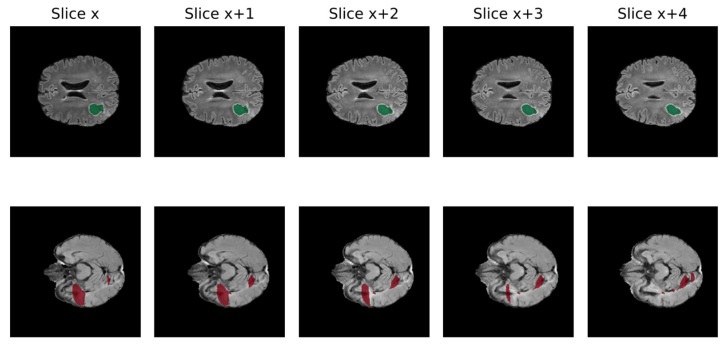
Qualitative comparison of processed FLAIR from volumetric raw input (above) and non-volumetric one in 5 different slices of the same MRI (Green: cavity, Red: FLAIR hyperintensity) [46].

A significant obstacle to the efficient growth of AI application in clinical practice is data quality. AI models require large amounts of high-quality images to obtain reliable results, whereas medical data frequently present suboptimal qualities for this task [61]. In fact, suboptimal quality of data is very common in clinical practice, including non-volumetric scans, missing sequences, and artifacts [62]. Low quality may negatively impact the performance because real inter-image variability results are hardly distinguishable from artifacts. In Figure 3, the non-volumetric image negatively affected the result of the segmentation from a DL-based algorithm [47].

Thus, problems such as bias, improper curation, and low reliability could be introduced [63]. Bias might occur when the AI models are trained on data that are not representative of the target patient population. In particular, DL algorithms are often trained on cured and standardized datasets that do not represent clinical data heterogeneity and quality. Although this selection bias makes the training process easier, it makes the results not as easily transferable to real-world clinical practice [61].

Idealistically, the elimination of non-volumetric scans and low-quality imaging from clinical practice would have the greatest impact for the future clinical application of AI technologies. Nevertheless, at present, it is essential to train the models to perform adequately despite the heterogeneity and the complexity of the cases. Recent efforts from BraTS are aimed at including imaging acquired with lower technologies, such as MRIs from Sub-Saharan Africa [64]. This choice pushes the efforts towards advanced image preprocessing to enhance the resolution and other tools able to support the most accurate segmentation, even in complex scenarios [65].

### 3.3. Data Selection

The results obtained from most of the algorithms are not easily reproducible in the real world context since they are frequently trained on curated and standardized datasets that do not include suboptimal-quality images. Although this selection bias makes the training process easier, it is not as easily transferable to real-world clinical practice. In fact, suboptimal quality of data is very common in clinical practice, including non-volumetric scans, missing sequences, and artifacts [66]. Moreover, the current literature often presents strong criteria for the inclusion/exclusion of MRI scans in the final datasets. For example, regarding postoperative brain tumor segmentation, criteria generally concern the exclusive inclusion of newly diagnosed GBM, the availability of all imaging modalities, or the defined presence of the resection cavity on visual inspection. An additional selection bias for studies concerning DL methods for the segmentation of glioma MRIs concerns the diffused use of publicly available benchmarks, such as BraTS and TCIA databases for the training phase [52]. On the one hand, their use has a positive role in the development of DL systems, but there is a substantial risk of overfitting. This could explain the high accuracy and reproducibility obtained by different reported algorithms [52].

In a recent study, a DL algorithm was trained on an MRI database that is representative of the real-world scenario, thus including heterogeneous and incomplete data [46]. In this study, inclusion criteria were not restrictive concerning the quality of the available data to avoid selection bias. So, low-quality images (e.g., non-volumetric imaging) and incomplete cases (with missing sequences) were also included. Notably, there is not a benefit in performance from incorporating non-volumetric imaging since its inclusion in the dataset creates a difficult scenario for the algorithm to be correctly classified. Indeed, the benefits resulting from the incorporation of these data are related to clinical applicability of the algorithm. Moreover, an increased heterogeneity of data would derive from a multi-institutional cooperation to create a real clinical database. In fact, having images acquired with different protocols, resolution, and contrast (1.5 T or 3 T) would make the learning process more complex but would probably result in increased adaptability of the algorithm to different clinical scenarios. It is necessary to point out that the positive role of heterogeneity of data for the training phase depend on the final purpose of the algorithm. In case the application concerns a specific scenario with defined features required for the dataset, heterogeneity may not have a positive impact.

Concerning machine learning algorithms which can identify gliomas in datasets containing non-glioma images, some studies have recently been performed in this regard but the algorithms should be further developed to allow for integration into clinical workflow [67].

### 3.4. Focus on Preoperative Scenario

**Figure 4:**
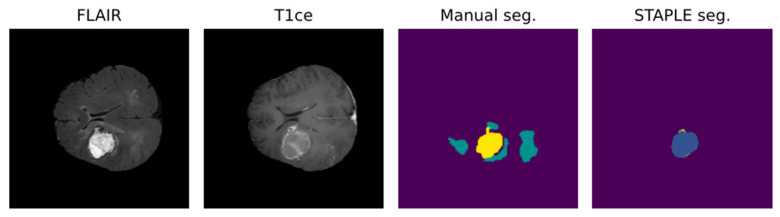
DL-based postoperative segmentation (STAPLE seg.) still obtains non-accurate results in some cases, as the one presented above [46]. In this example, the DL result completely diverged from the manual segmentation.

Notably, most algorithms in the current literature are based on preoperative tumor imaging, whereas most clinical imaging techniques for brain tumors are used after treatment to assess response or to monitor progression [52]. Limitations in postoperative MRI evaluation are partly due to artifacts, caused by blood and air in the resection cavity (RC), and logistical issues in collecting data regularly from the same patient during follow-up [68,69]. Particularly, the RC is frequently a source of artifacts in the MRI because of blood residuals and air bubbles [58,70]. In addition to this, brain anatomy may be partly altered because of the surgical act, the post-surgical edema, and the tumor itself [70]. These problems lower the accuracy of available algorithms in obtaining a postoperative evaluation of MRI. Figure 4 shows an example of the misclassification of postoperative MRI obtained by a reliable DL tool for MRI pre- and postoperative segmentation [46].

Moreover, postoperative images have different acquisition times given the time-course of the disease and the treatment schedule. This means that the postoperative MRI database contains images from different points in time: immediate postoperative, before and after adjuvant treatment, and regular follow-up. Recently, some studies reported good accuracy in postoperative segmentation of MRI, though it is still far from the level of accuracy achieved in preoperative evaluation [66,71]. Moreover, the absence of a wide dataset like BraTS requires researchers to deploy their model on small private collections, hence reducing comparability and generalizability.

The future perspective includes the creation of multi-institutional databases including postoperative MRIs. Still, due to the complex nature of such a task, some studies have tried to mimic postoperative MRIs by exploiting generative adversarial network (GAN) capabilities in synthesizing fake-yet-plausible MRIs starting from real scans. A recent study from the State Key Laboratory of Oncology in South China [72] proposed CoCosNet, a neural network showing interesting results in artificially synthesizing postoperative weighted-T1 MRIs from the corresponding preoperative ones and postoperative CTs.

Considering the data-intensive nature of deep DL solutions, the predominant recommendation continues to be the collection of multimodality and multi-institutional MRI data. This strategy aims to normalize and synchronize the postoperative scenario in a manner analogous to how the BraTS challenge has standardized the preoperative phase.

## 4. Molecular Subtyping

Many recent research articles have reported remarkable success in the use of artificial intelligence to predict the status of 1p19q codeletion, IDH1 mutation, and MGMT promoters. These molecular features acquired an increasing interest as they are related to the prognosis and to the identification of the best treatment options for each singular patient [73]. Also, the BraTS challenge recently gave more relevance to these features. In fact, the second task of BraTS 2021 consisted of the evaluation of methods to predict the MGMT promoter methylation status.

### 4.1. IDH Mutation

Having IDH1 or IDH2 mutations is associated with improved survival [74,75] as these gliomas respond better to temozolomide therapy [76]. IDH-mutant gliomas demonstrate lower regional cerebral blood volume and flow on MR perfusion, higher apparent diffusion coefficients on diffusion MR imaging, and improved survival [77,78]. In a study by Beiko et al. [79], the resection of non-enhancing gliomas correlated with improvements in PFS in HGG with mutated IDH gliomas as opposed to IDH wild-type tumors. Thus, the knowledge of IDH mutation status before surgical resection may be important. The features that mattered most to predict IDH mutation status include absent or minimal enhancement, central areas with low T1 and FLAIR signal and well-defined tumor margins according to Liang et al. [80]. Their study was performed using the publicly available BraTS 2017 database and finally achieved 84.6% accuracy [77]. More recently, the studies from Chang et al. [81] obtained accuracy levels higher than 90% for the prediction.

### 4.2. P/19q Codeletion

Indeed, there are a paucity of manuscripts using CNNs to predict 1p19q codeletion. In one of the most successful studies, Chang et al. [81] succeeded in predicting 1p19q codeletion status with an accuracy of 92%. They employed the component analysis to define the features that were mostly related to the codeletion. According to their analysis, the 1p19q codeletion status is related to frontal lobe location, ill-defined tumor borders, and larger amounts of contrast.

### 4.3. MGMT Methylation

The hypermethylation of the MGMT promoter is strongly associated with better response to temozolomide chemotherapy and improved prognosis [82,83]. Nevertheless, the prediction of MGMT mutation status from preoperative MRI using AI has achieved modest results by now [84]. More recent studies from Korfiatis et al. [85] and Chang et al. [86] obtained accuracies higher than 80% for the prediction of MGMT status. Again, in this paper, the use of principal component analysis for dimensionality reduction determined that the most important imaging features for the prediction of MGMT status included heterogeneous and nodular enhancement, the presence of eccentric cysts, more mass-like T2/FLAIR signal with cortical involvement, and frontal/temporal lobe locations [86]. These findings confirm the results from prior MR genomics studies [85,87].

In summary, molecular features such as IDH mutation, 1p19q codeletion, and MGMT promoter status are successfully predicted by AI applied to MRI. Moreover, algorithms obtaining accuracies of prediction exceeding 80% to 90% may probably already be superior to human-level performance. This expanding field could have a further impulse and improvement in performance with new coder architecture, large-scale data sharing, and integration of clinical data.

## 5. Ethical Concerns

### 5.1. Lack of Standard Guidelines for Clinical Studies

The advent of deep neural networks has engendered many applications in medical imaging [88]. Currently, the field of radiomics lacks standardized evaluation concerning both scientific integrity and clinical relevance of the published radiomics investigations [89]. Rigorous evaluation criteria and reporting guidelines need to soon be established and rigorously respected to obtain clinical applicability [90].

To guarantee a high standard of research and to obtain reproducible results, radiomic investigations should respect the well-defined criteria of reliability and reproducibility concerning both the presented results and the applied methods. Numerous checklists have recently been proposed and came into widespread use [52]. Those checklists include the Standards for Reporting of Diagnostic Accuracy Studies (STARD) [91,92], Strengthening the Reporting of Observational studies in Epidemiology (STROBE) [93], and Consolidated Standards of Reporting Trials (CONSORT) [94,95]. The most recent STARD list was released in 2015 and included 30 items identified by an international group of methodologists, researchers, and editors. Those items were identified so that, when they reported, readers can judge the potential bias in the study, appraise the applicability of the study findings and the validity of the conclusions. Indeed, the Checklist for AI in Medical Imaging (CLAIM) is modeled after the STARD guideline, but it has been extended to address applications in classification, image reconstruction, text analysis, and workflow optimization. These elements are considered as “best practice” elements that should guide authors in presenting their research [96].

### 5.2. Lack of Transparency

Interpretability of an AI program is defined as the human ability of understanding the link between the initial features extracted by the program and the final prediction obtained. As DL structures are typically complex and composed of numerous hidden layers, this link is difficult to find. This concept is commonly referred to as the “black-box problem” [97]. Nevertheless, the lack of transparency regarding AI techniques is a significant concern. Any medical care system needs to be understandable and explicable for physicians, administrators, and patients. It should ideally be able to fully explain the reasoning behind a decision to all parties concerned [61].

Interpretability methods are approaches designed to explicitly enhance the interpretability of a machine learning algorithm, despite its complexity [97].

Interpretability techniques like guided backpropagation, gradient-weighted class activation mapping (Grad-CAM) [98], and regression concept vectors [99] are being applied to medical images, illustrating the burgeoning interest in Explainable AI (XAI) and Neuro-symbolic AI (NeSymAI). These fields are gaining traction as they provide crucial insights into the reasoning behind AI models, particularly in the medical realm, making the interpretation of predictions vital [100,101]. The repertoire of interpretability methods is expanding to keep pace with the complexity of radiology practices, which increasingly integrate different types of patient data, such as imaging, molecular pathways, and clinical scores Hence, interpretability approaches capable of processing this diverse information are seen as highly promising [102].

Also, interdisciplinary collaboration may limit the lack of transparency and, thus, support the acceptance of AI in clinical practice [61]. Surgeons, radiologists, data scientists, AI experts, and ethicists must collaborate to create robust guidelines and standards for the ethical deployment of AI tools in patient care. Moreover, the relationship with the patient plays an essential role in this process of acceptance [60]. In fact, the patients deserve to understand how AI influences their medical care and outcomes. For this reason, a careful and complete informed consent should not be underestimated for the perspective introduction of AI into clinical practice.

### 5.3. Privacy and Data Protection

Until the past decade, medical imaging included 2D images. However, advances in imaging methods have made high-resolution 3D imaging a reality through smoothing, interpolation, and super-resolution methods, enabling accurate volume rendering [103]. With advances in facial recognition, it is not difficult to match images generated from CT or MRI scans to photographs of an individual. For this reason, in medical imaging research, it is standard practice to modify images using defacing or skull-stripping algorithms to remove facial features [61]. Additionally, this process reduces inter-patient physiological variability, and the pathological aspects can emerge more relevantly. Unfortunately, such modifications can negatively affect the generalizability of machine learning models developed using such data [103]. Anyway, patient privacy is a major health system concern requiring multiple legal quandaries to be addressed prior to uploading and diffusing data [37].

Moreover, when considering advanced healthcare imaging, it is difficult to obtain a complete anonymization, despite the efforts in data protection. The basic task seems straightforward: selectively remove or codify identifiers in the metadata header content of images. Although nearly all radiologic data use a universal format, DICOM, there are a growing number of exceptions, making it more difficult to standardize processes.

A balanced solution likely involves making information about AI systems and data collection understandable for patients, creating relationships of trust between institutions and their patients. At the same time, the aim should be to obtain more effective deidentification models that reduce identifiability as complete anonymization does not seem possibly obtainable in the near future. Current best practices for deidentification in radiology include avoiding the placement of identifiable data in proprietary DICOM fields, optimizing protocols of data management, using validated and tested protocols for deidentification, and investigating safer means of data sharing, such as containerization and blockchain [61]. There are calls for the formation of advisory committees to periodically review the protocols concerning privacy issues and identifiability in imaging [103]. As an example, skull-stripping—also known as brain extraction consisting of the act of removing non-brain signal from MRI data—is usually performed not only to remove redundant information, but especially to avoid facial reconstruction and identification.

## 6. Conclusions

Deep learning has provided reliable results for GBM assessment concerning MRI analysis and segmentation, including molecular, prognostic, and diagnostic information. We extensively reviewed the issues currently limiting its routine clinical application. Nevertheless, these limitations could be prospectively addressed in the near future. The most important matters concern the introduction and development of new technical advancements, an increased attention to data collection, and the careful addressing of healthcare ethical issues.

## Figures and Tables

**Table 1:** Summary of the presented concerns regarding AI application for glioblastoma MRI segmentation. Each limitation is accompanied by the domain of pertinence, the definition of the problem, and the proposed solution(s).

Section	Limitation	Domain	Definition	Possible Solution(s)
2.1	imaging heterogeneity	technical	scanner-dependent variation in image signal intensity	intensity standardization
				rescanning data
2.2	missing MRI sequences	technical	unavaiable modality/ies (T1, T2, FLAIR, T1CE)	inter-modality translation
				knowledge distillation
2.3	deployment issues	technical	limited computational resources and memory constraints	tiling
				quantization
2.4	performance evaluation	technical	subjective reference standards	cross-validation
				unsupervised training
3.1	limited number of patients	application	low number of data publicly avaiable	transfer learning
3.2	data quality	application	suboptimal quality of data (non-volumetric scans)	pre-processing
				inclusion of complex scenarios
3.3	data selection	application	selection bias and reduced applicability	inclusive database
3.4	focus on preoperative scenario	application	logistical and technical issues for postop. MRIs	multi-modality and multi-institutional data
4	exclusion of molecular data	molecular	limited consideration of IDH—1p/19q—MGMT	new coder architecture
				large-scale data-sharing
5.1	lack of standard guidelines	ethical	scientific integrity not definable	checklist
5.2	lack of transparency	ethical	limited understanding of the results	interpretability methods
				interdisciplinary collaboration
5.3	privacy and data protection	ethical	difficulty to obtain complete anonimization	skull-stripping
